# Supplementary figures and images for: Contrasting Roles of Programmed Death-Ligand 1 Expression in Tumor and Stroma in Prognosis of Esophageal Squamous Cell Carcinoma
Source: Cancers (Basel). 2024 Mar 13;16(6):1135. doi: 10.3390/cancers16061135 (PMC10969310; doi:10.3390/cancers16061135)

Figure S2

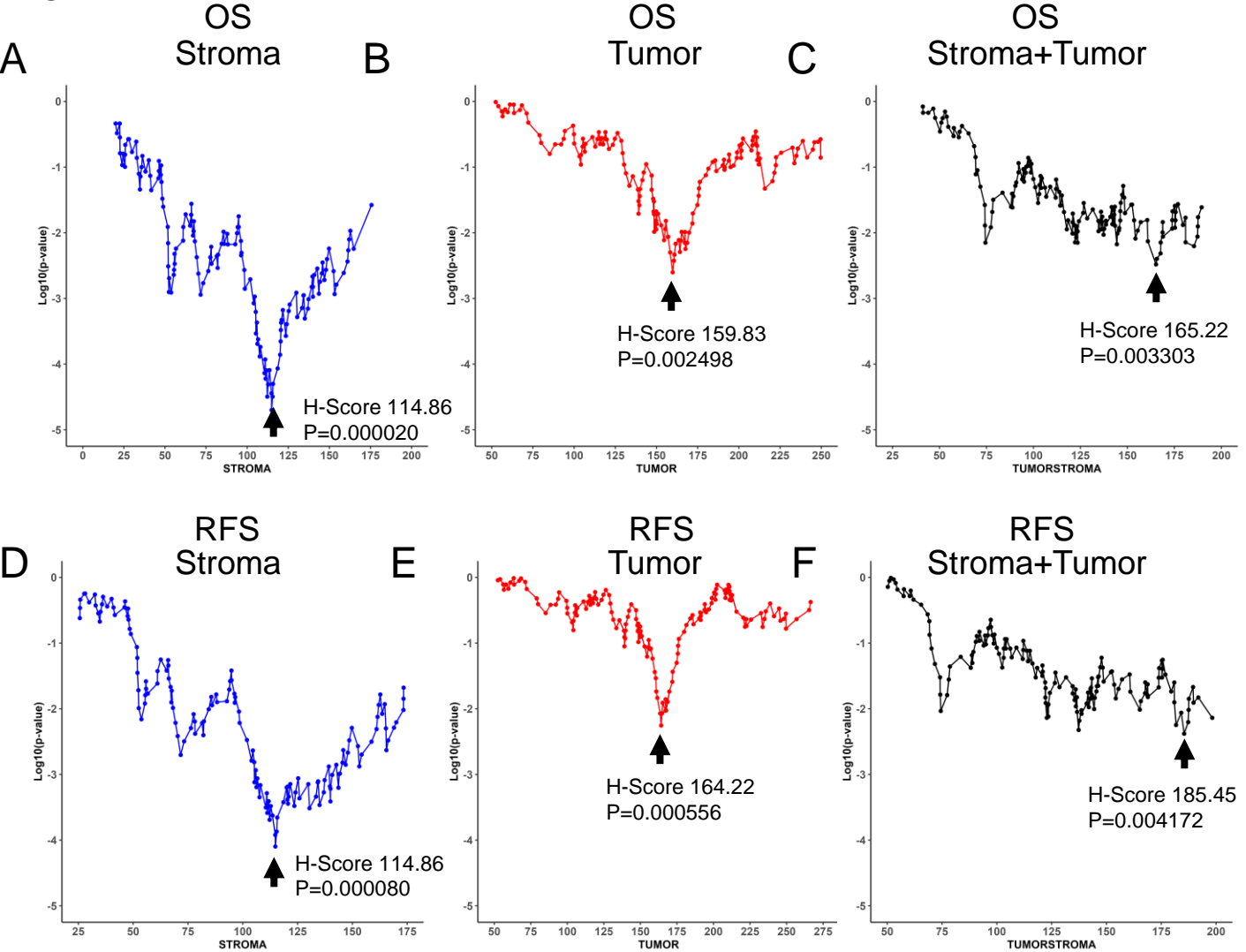

Supplement: Supplementary file 1 [file cancers-16-01135-s001.zip › Figure S2.pdf]

Figure S1

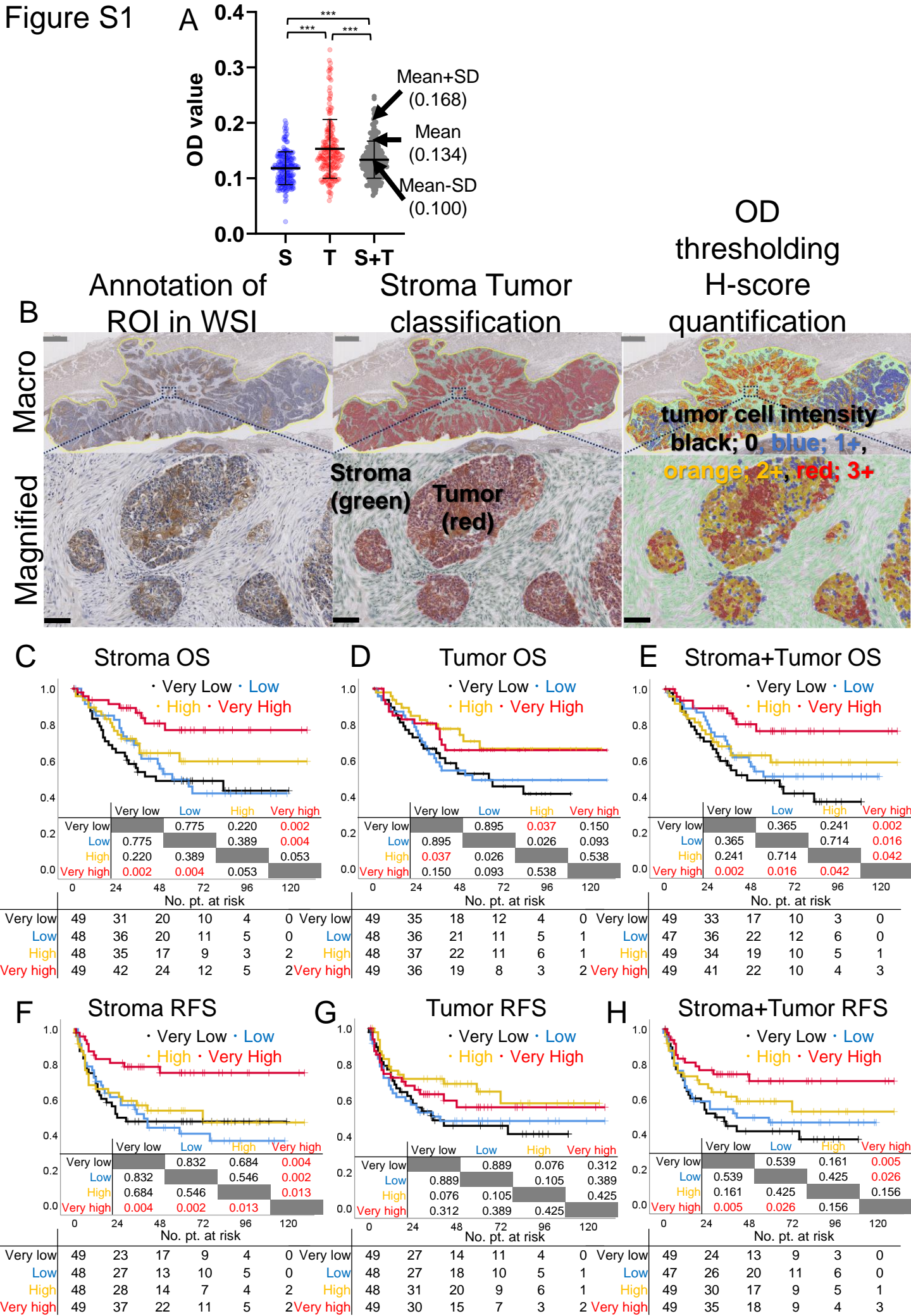

Supplement: Supplementary file 1 [file cancers-16-01135-s001.zip › FigureS1.pdf]
